# Epsti1 Regulates the Inflammatory Stage of Early Muscle Regeneration through STAT1-VCP Interaction

**DOI:** 10.7150/ijbs.94675

**Published:** 2024-06-24

**Authors:** Jee Won Kim, Ju-Hyeon Bae, Ga-Yeon Go, Jae-Rin Lee, Yideul Jeong, Jun-Young Kim, Tae Hyun Kim, Yong Kee Kim, Jeung-Whan Han, Ji-Eun Oh, Myong-Joon Hahn, Jong-Sun Kang, Gyu-Un Bae

**Affiliations:** 1 Drug Information Research Institute, Sookmyung Women's University, Seoul 04310, South Korea.; 2 Muscle Physiome Research Center, Sookmyung Women's University, Seoul 04310, South Korea.; 3 College of Pharmacy, Sookmyung Women's University, Seoul 04310, South Korea.; 4 Department of Molecular Cell Biology, Sungkyunkwan University School of Medicine, Suwon 16419, South Korea.; 5 Cell and Gene Therapy Products Division, National Institute of Food and Drug Safety Evaluation, Cheongju 28159, South Korea.; 6 Research Institute of Aging Related Disease, AniMusCure Inc., Suwon 16419, South Korea.; 7 Research Center for Epigenome Regulation, School of Pharmacy, Department of Biochemistry and Molecular Biology, Sungkyunkwan University, Suwon 16419, South Korea.; 8 Department of Biomedical Laboratory Science, Far East University, 76-32 Daehakgil, Gamgok-myeon, Eumseong-gun, Chungbuk-do, 27601, Korea.

**Keywords:** Muscle regeneration, Inflammatory response, IFN-*γ*-JAK-STAT1 pathway, Ubiquitin-proteasomal degradation, VCP, Epsti1

## Abstract

During muscle regeneration, interferon-gamma (IFN-*γ*) coordinates inflammatory responses critical for activation of quiescent muscle stem cells upon injury via the Janus kinase (JAK) - signal transducer and activator of transcription 1 (STAT1) pathway. Dysregulation of JAK-STAT1 signaling results in impaired muscle regeneration, leading to muscle dysfunction or muscle atrophy. Until now, the underlying molecular mechanism of how JAK-STAT1 signaling resolves during muscle regeneration remains largely elusive. Here, we demonstrate that epithelial-stromal interaction 1 (Epsti1), an interferon response gene, has a crucial role in regulating the IFN-*γ*-JAK-STAT1 signaling at early stage of muscle regeneration. Epsti1-deficient mice exhibit impaired muscle regeneration with elevated inflammation response. In addition, Epsti1-deficient myoblasts display aberrant interferon responses. Epsti1 interacts with valosin-containing protein (VCP) and mediates the proteasomal degradation of IFN-*γ*-activated STAT1, likely contributing to dampening STAT1-mediated inflammation. In line with the notion, mice lacking Epsti1 exhibit exacerbated muscle atrophy accompanied by increased inflammatory response in cancer cachexia model. Our study suggests a crucial function of Epsti1 in the resolution of IFN-*γ*-JAK-STAT1 signaling through interaction with VCP which provides insights into the unexplored mechanism of crosstalk between inflammatory response and muscle regeneration.

## Introduction

Skeletal muscles, which constitute about 40-50 % of total body weight in humans, have a resilient regeneration capacity in response to injury and defects in regeneration capacity have been implicated in muscle wasting related to aging and degenerative diseases 1. Muscle regeneration is a highly coordinated multistep process orchestrated by various cellular and molecular responses, including degeneration/necrosis, inflammation, myogenesis, remodeling, and maturation 2. Inflammation, the early stage of the regeneration process, is responsible for the initiation of myogenesis by activating quiescent muscle stem cells and shifting their proliferative state into differentiating state 3, 4. However, excessive or persistent proinflammatory responses cause perturbation in the progression of muscle stem cell states, leading to impaired muscle regeneration and eventually contributing to muscle dysfunction or muscle atrophy 5-7.

Among several inflammatory cytokines, interferon-gamma (IFN-*γ*), being increased within the first 24 hours following muscle injury, is crucial for coordinating the inflammatory response with myogenesis 3, 8. IFN-*γ* activates the Janus kinase (JAK) - signal transducer and activator of transcription (STAT1) pathway in macrophages, promoting pro-inflammatory M1 phenotypes 9. It also activates JAK-STAT1 signaling in myogenic precursor cells (MPCs), maintaining their proliferative and non-differentiated state to facilitate population expansion for the efficient repair 10, 11. However, despite the crucial role of IFN-*γ* at the early regeneration, JAK-STAT1 signaling at the later stages inhibits muscle regeneration 11-13. Therefore, it is crucial to gain an insight into the molecular mechanisms controlling IFN-*γ*-JAK-STAT1 activation and suppression in the stage progression of muscle regeneration. One likely mechanism implicated in signal suppression is the degradation of phosphorylated STAT1 by the ubiquitin-proteasome system (UPS) 14, however, the modulatory mechanisms leading to suppression of IFN-*γ*-JAK-STAT1 signaling in muscle regeneration are largely unknown.

Epithelial-stromal interaction 1 (EPSTI1) is first identified through its increased gene expression in breast cancer epithelial cells when cocultured with stromal fibroblasts and is known as an interferon response gene. 15. Recent studies have shown that EPSTI1 is highly increased in patients having inflammatory diseases (systemic lupus erythematosus, lymphadenitis, and COVID-19) or cancers 16-19. Although the molecular function of Epsti1 has rarely been investigated, a study suggested that Epsti1 can interact with valosin-containing protein (VCP), facilitating ubiquitin-proteasome-dependent protein degradation 20.

In the current study, we investigated the role of Epsti1 in muscle regeneration. EPSTI1 is upregulated in muscle and muscle stem cells (MuSCs) at the early stage of regeneration. Mice lacking Epsti1 exhibited impaired muscle regeneration with elevated inflammatory responses. Furthermore, Epsti1-deficient myoblasts exhibited decreased myogenic potential accompanied by increased STAT1 signaling. The mechanistic study revealed that Epsti1 interacts with VCP and mediates the proteasomal degradation of IFN-*γ*-activated STAT1. Furthermore, in a cancer-cachexia model, Epsti1 deficiency resulted in exacerbated muscle wasting accompanied by excessive inflammation. In summary, our findings demonstrate a novel function of Epsti1 in resolving inflammatory responses and augmenting myogenic progression, thereby contributing to robust muscle regeneration.

## Methods

### Animal experiments

The protocols of animal experiments were reviewed and approved by the Institutional Animal Care and Use Committee of Sookmyung Women's University and carried out by the ethical guideline (the protocol number: SMWU-IACUC-2005-003-01). Epsti1^-/-^ (Epsti1 KO) were obtained by breeding Epsti1^+/-^ pairs and for all experiments we have used the littermates with the genotype of Epsti1^+/+^ (WT) and Epsti1^-/-^ as previously described 21. The primer sequences for genotyping were listed in Table S1.

To evaluate skeletal muscle regeneration, 16-20-week-old male mice were anesthetized with 1%-2% isoflurane. Tibialis anterior (TA) muscles were injected with 20 μM of cardiotoxin (Sigma, USA) in a volume of 50 μl. Mice were then sacrificed at 0, 2, 4, 7, 14, and 21 days after injury to assess the regeneration process.

To induce cachexia, 16-20 weeks old male mice were anesthetized with 1-2% isoflurane. Mice were subcutaneously injected with LLC cells (5 × 10^6^)/PBS (100 μl) or PBS alone into the flank. Mice were then sacrificed 30 days after injection to examine the development of cachexia.

### Isolation of primary myoblasts

Primary myoblasts were isolated from hind limb muscles of WT and Epsti1 KO mice aged 2-3 weeks. Muscles were dissected and digested with collagenase/dispase (Roche, Switzerland). Isolated cells were plated on collagen-coated dishes and cultured in F-10 medium (Gibco, USA) containing 20% fetal bovine serum (Gibco, USA), 100 units/ml penicillin and 100 μl/ml streptomycin (Welgene, South Korea) in the presence of 2.5 ng/ml basic FGF (Gibco, USA) and 10 ng/ml HGF (Millipore, Germany). To induce myogenic differentiation, primary myoblasts were exchanged into differentiation medium, high glucose Dulbecco's modified Eagle's medium (DMEM) (Gibco, USA) containing 2% horse serum (Life Technologies, USA), 100 units/ml penicillin and 100 μl/ml streptomycin.

### Cell culture and reagents

C2C12 and HEK293T cell lines were obtained from American Type Culture Collection. C2C12 myoblasts were cultured in the growth medium, high glucose DMEM (Gibco, USA) containing 15% fetal bovine serum (Gibco, USA), 100 units/ml penicillin-100 μl/ml streptomycin (Welgene, South Korea). To induce myogenic differentiation, 80%-90% confluent C2C12 cells were exchanged into differentiation medium, high glucose DMEM containing 2% horse serum (Life Technologies, USA), 100 units/ml penicillin -100 μl/ml streptomycin (Welgene, South Korea). HEK293T cells were cultured in high glucose DMEM containing 10% fetal bovine serum, 100 units/ml penicillin-100 μl/ml streptomycin. All cells were maintained at 37℃ under 5% CO2 in an incubator. Recombinant mouse interferon-gamma protein (R&D, USA), MG-132 (Sigma, USA), and NMS-873 (Sigma, USA) were prepared according to the manufacturer's instructions.

### Constructs

Deletion mutant constructs of Epsti1 were cloned into a pCMV vector containing an N-terminal SRT tag 21. Detailed designs of the cloning strategy for each construct are illustrated in Figure 4D. Mouse VCP gene was amplified by RT-PCR of mRNAs purified from C2C12 myoblasts and verified by sequencing followed by cloning into a pcDNA3.1 vector containing an N-terminal Myc tag (Addgene, USA).

### Epsti1 knockdown using lentiviral shRNA

For shRNA lentivirus generation, pLKO.1-shNC (SHC002) or pLKO.1-shEpsti1 (TRCN0000192152) (Sigma, USA) was used. HEK293T cells were transfected with these lentiviral vector plasmids (psPAX2) and packing plasmids (pMD2.G) (Addgene, USA) using jetPEI (Polyplus, France). After transfection, the supernatant containing lentivirus was collected, filtered (0.45μm), and concentrated using a Lenti-X concentrator (TAKARA, Japan). The aliquots were stored at -80℃. The titer of the lentiviral supernatant was measured by HIV-1 p24 Elisa assay (Origene, USA). C2C12 cells were transduced with MOI 200 using 8 μg/ml polybrene and selected with 2 μg/ml puromycin for 3 days.

### Immunoblot and immunoprecipitation analysis

Whole-cell extracts were obtained by RIPA buffer supplemented with 10 mM sodium fluoride, 1 mM sodium vanadate, and 1x protease inhibitor (Gendepot, USA). Cell extracts were sonicated and centrifuged to clarify lysates. Protein concentration was determined by BCA assay (Thermo Fisher, USA) according to the manufacturer's instructions. After boiling in the sample buffer, an equal amount of protein lysates were subjected to SDS-PAGE, followed by transfer to PVDF membranes. The membranes were blocked with 5% skim milk and incubated with primary antibodies. Next, the membranes were incubated with HRP-linked secondary antibodies and the signals were detected using ECL substrate (Abclon, South Korea). Antibodies used for immunoblot analysis were listed in Table S2.

Preparation of cell lysates for immunoprecipitation was performed as preparation for immunoblot analysis. After protein extraction, appropriate antibodies were added, followed by Protein A/G agarose beads (Roche, Switzerland). Complexes were eluted and analyzed by SDS-PAGE and immunoblotting. Antibodies used for immunoprecipitation were listed in Table S2.

### Immunohistochemistry and Immunofluorescence staining

Freshly dissected muscles were frozen in OCT and cryosectioned with 7 μm thickness.

For histological analysis of TA muscles, cryosections were fixed using 4% paraformaldehyde and stained with Harris' hematoxylin (Sigma, USA) and eosin (BBC Biomedical, USA) according to the manufacturer's protocol.

For immunofluorescence staining of TA muscles, fixed cryosections were permeabilized using 0.1% triton X-100 (Sigma, USA), followed by antigen retrieval. After blocking with 5% goat serum (Thermo Fisher, USA), they were immunostained with primary antibodies. Next, the TA muscles were incubated with fluorochrome-linked secondary antibodies. Nuclei were counterstained with DAPI (Sigma, USA). Antibodies used for immunofluorescence staining were listed in Table S2.

For immunostaining for cells, fixation, permeabilization, blocking, and incubation of antibodies were processed as immunostaining for muscles. Nuclei were counterstained with Hoechst (Thermo Fisher, USA). Antibodies used for immunofluorescence staining were listed in Table S2.

Images were acquired by Axio Observer Z1 or LSM-700 (Zeiss, Germany), and analyzed by using ImageJ or ZEISS Zen Imaging Software.

### Quantitative RT-PCR analysis

Total RNA was extracted from muscles and cells using Trizol (Invitrogen, USA) according to the manufacturer's instructions, then cDNA was synthesized by iScript reverse transcriptase (Bio-Rad, USA). qRT-PCR analysis performed with SYBR green (Applied Biosystems, USA) and listed primers in Table S1. The transcript levels of each gene were normalized to the level of GAPDH.

### Gene set enrichment analysis (GSEA) and Single-cell RNA-seq (scRNA seq) data analysis

The unbiased Gene Set Enrichment Analysis was performed using GSEA software (Broad Institue; software.broadinstitute.org/gesa/). The microarray dataset was derived from the Gene Expression Omnibus database (GSE45577).

All scRNA-seq datasets were analyzed using the SeqGeq^TM^ (three stars) program. The datasets were integrated using the pipeline of the Dimensionality Reduction platform. Integrated cells underwent a quality control process to remove dead cells and doublets, which was based on library size and cells expressing dispersion. Subsequently, the integrated cells were normalized as Counts per Million (CPM). The filtered cells were then clustered using the Seurat pipeline with a resolution of 0.5. To visualize the data, a dimensional reduction UMAP was generated through the Seurat function RunUMAP. Cluster identities were assigned based on top marker genes and the expression of known marker genes from published literature. For the MuSC (Muscle Stem Cell) clusters, a dimensional reduction UMAP was generated using the Seurat function RunUMAP to assess the myogenesis status. The differentiation of MuSCs was analyzed using the monocle-based trajectory workflow.

### Statistical analysis

All data were presented as mean ± standard deviation (S.D.) or standard error of the mean (S.E.M.), as indicated in the figure legends. GraphPad Prism 10 software was utilized for all statistical analyses. For comparisons between the two groups, statistical significance was evaluated using an unpaired two-tailed Student's t-test. For comparisons among multiple groups, statistical significance was evaluated using one-way ANOVA follows by the Dunnett post hoc test or two-way ANOVA followed by the Sidak post hoc test. Statistical significance was indicated as *P < 0.05, **P < 0.01, *** P < 0.001, ****P < 0.0001, ns not significant.

## Results

### Epsti1 is upregulated in early stages of muscle regeneration

**Figure 1:**
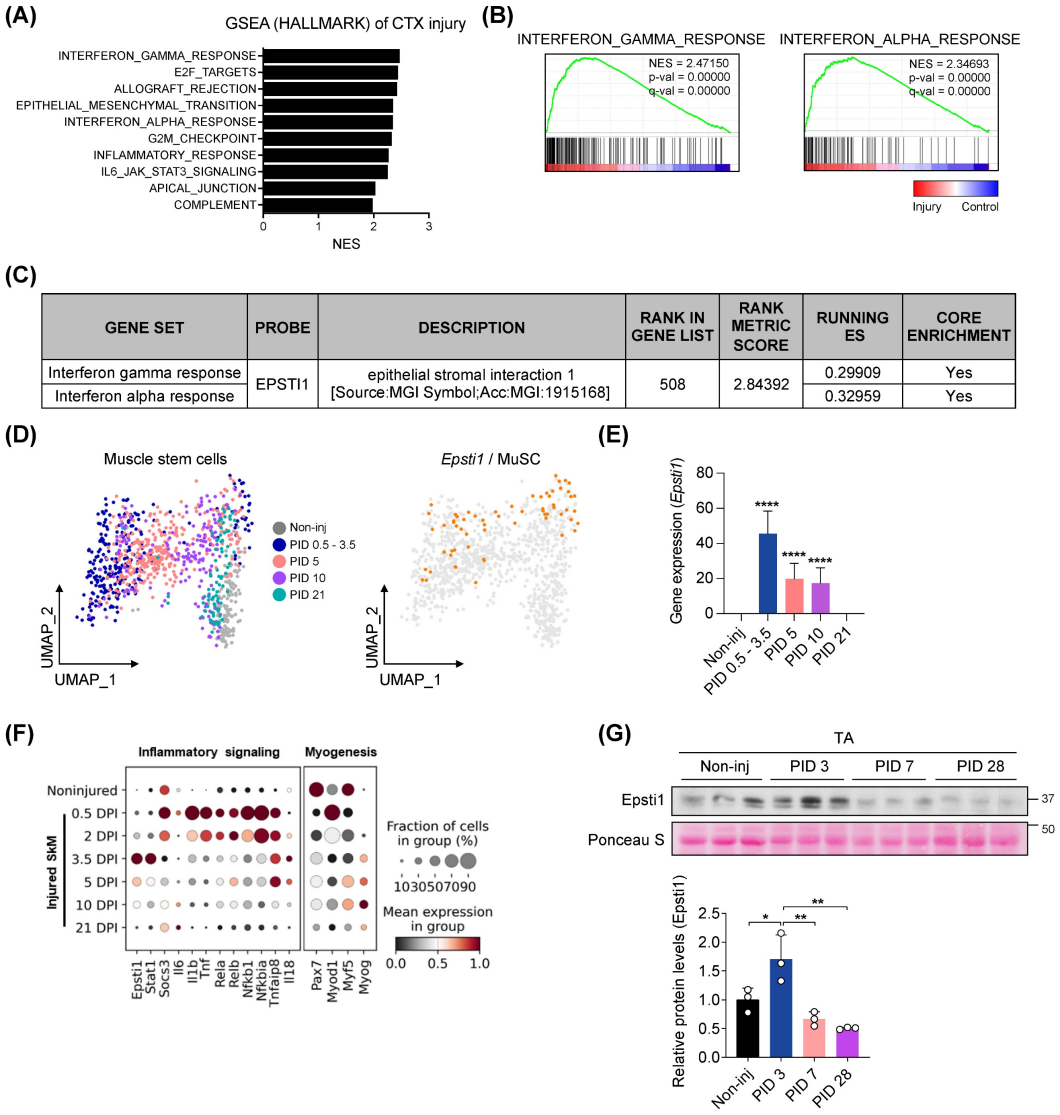
***Epsti1* is upregulated at the early stage of muscle regeneration.** (A) Top 10 Hallmark biological processes enriched in muscles at 3 days after cardiotoxin (CTX) injury. (B) Enrichment plots of interferon-gamma response and interferon alpha response gene set in CTX-injured muscles. (C) Information that Epsti1 belongs to the leading subset of the interferon-gamma and interferon alpha response gene sets in CTX-injured muscles. (D) UMAP embedding of muscle stem cells (MuSCs) during muscle regeneration at indicated post injury day (PID). The expression of Epsti1 is colored in orange dots; all other cells are colored in gray (GSE138826). (E) Bar graph showing the expression of Epsti1 during muscle regeneration. (F) Dot plots showed Epsti1, inflammatory signaling-, and myogenesis-related genes after days post-injury (DPI). (G) Immunoblot analysis of Epsti1 in tibialis anterior (TA) muscles at the indicated day of PID. Staining intensity of Poncuea S was serves as a loading control. Below graph indicates relative Epsti1 protein levels, *n* = 3 mice per group. In (E) and (G), data are presented as mean ± S.D. The statistical analysis was performed using one-way ANOVA. ****p<0.0001.

To assess changes in gene sets associated with the early stage of muscle regeneration, we performed an unbiased gene set enrichment analysis (GSEA) using publicly available microarray data (GSE45577). We analyzed the data from injured muscles at 3 days post-injury (PID 3) induced by either cardiotoxin (CTX) (Figure 1A-C) or glycerol (Figure S1A-C). Out of the 49 gene sets categorized under the Hallmark biological process, 21 gene sets were significantly (FDR < 25%, p-value < 1 %) enriched in the injured group. Most of the top 10 gene sets, ranked by their normalized enrichment score (NES), were related to the inflammatory response (Figure 1A). Consistent with previous studies 16-19, 22, Epsti1 was involved in both IFN-*γ* and *α* response gene sets (Figure 1B). Moreover, *Epsti1* was included in the leading-edge subset, a biologically significant subset within the interferon response gene sets (Figure 1C). In addition to the GSEA analysis of injured muscle, we evaluated the gene expression of *Epsti1* in MuSCs at different time points after injury, utilizing public single-cell RNA sequencing (scRNA-seq) data of MuSCs (GSE138826) (Figure 1D and E). We found that the gene expression of *Epsti1* in MuSCs increased sharply at 0.5-3 days post-injury and decreased over time as regeneration proceeded. Especially, an increase in Epsti1 was detected at 3 days post injury, and it increased after the expression of acute inflammatory genes such as tumor necrosis factor (Tnf) and interleukin 6 (Il6) (Figure 1F). Epsti1 proteins were also increased at PID 3 and then decreased at PID 14 and PID 28 (Figure 1G). Based on the increased expression of Epsti1 in both muscle and MuSCs during the early stage of muscle injury, we hypothesized that Epsti1 plays a regulatory role in the initial response to injury during muscle regeneration.

### Epsti1 deficiency causes impaired muscle regeneration with elevated inflammatory responses

**Figure 2:**
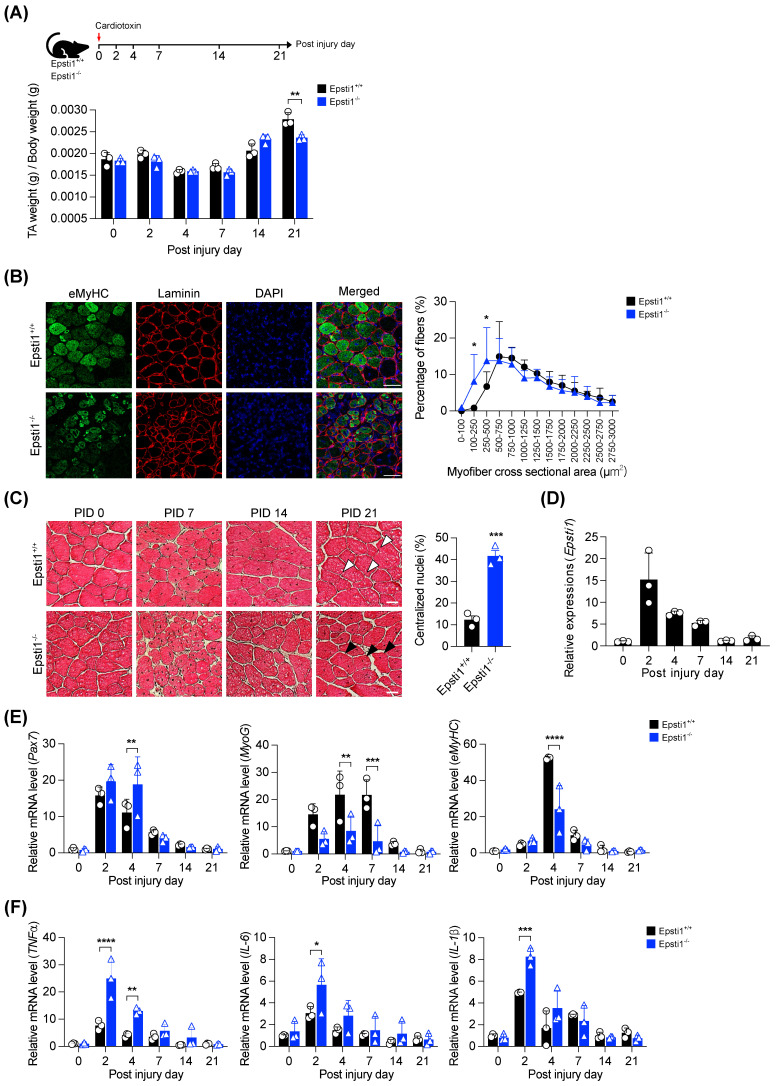
** Mice lacking Epsti1 exhibited impaired muscle regeneration with elevated inflammatory response.** (A) Experiment schematic of cardiotoxin (CTX) injury model and the ratio of TA muscle weight (g) per body weight (g) of Epsti1^+/+^ (WT) and Epsti1^-/-^ (Epsti1 KO) mice. (B) Representative images of immunostaining for eMyHC and laminin with TA sections from WT and Epsti1 KO mice at 7 days after CTX injury. Nuclei were stained with DAPI. Quantification of myofiber cross-sectional areas (CSA) in TA muscles of WT and Epsti1 KO mice is shown in the right panel. Scale bars, 50μm. (C) Representative images of H&E-stained TA sections from WT and Epsti1 KO mice at 0, 7, 14, and 21 days after CTX injury. White arrows indicate satellite cell nuclei on myofiber. Black arrows indicate centralized nuclei. Quantification of centralized nuclei is shown in the right panel. Scale bars, 30μm. (D) Relative mRNA levels of Pax7 from TA muscles at indicated PID. (E) Relative mRNA levels of Pax7, MyoG, and eMyHC from TA muscles of WT and Epsti1 KO mice at indicated day. (F) Relative mRNA levels of TNF*α*, IL-6, and IL-1*β* from TA muscles of WT and Epsti1 KO mice at indicated day. All data are presented as mean ± S.D. *n* = 3 mice per group. The statistical analyses were performed using two-way ANOVA (panel A, B, E and F), two-tailed Student's t-test (panel C) or one-way ANOVA (panel D). *p<0.05, **p<0.01, ***p<0.001, and ****p<0.0001.

To investigate the role of Epsti1 in muscle regeneration, we induced muscle injury in both Epsti1^+/+^ (WT) and Epsti1^-/-^ (Epsti1 KO) mice (Figure S2A and B). After 21 days of regeneration, we observed that the tibialis anterior (TA) muscle mass was significantly lower in Epsti1 KO mice than in WT mice (Figure 2A). Moreover, at 7 days post-injury, Epsti1 KO mice showed a lower number of newly regenerating myofibers that were smaller in size compared to WT mice (Figure 2B). Throughout the regeneration process, Epsti1 KO mice had smaller and more heterogeneous myofibers in size, compared to WT mice (Figure 2C). At 21 days of post-injury, control muscles had myofibers with laterally localized nuclei while myofibers of Epsti1 KO muscles still had mostly centralized nuclei. These findings suggest that Epsti1 deficiency results in delayed and impaired muscle regeneration.

We next analyzed the expression of critical regulators of muscle regeneration. Consistent with the GSEA and scRNA-seq analysis data, the expression of Epsti1 was significantly increased at the early stage of regeneration and gradually decreased as regeneration progressed (Figure 2D). In comparison to WT muscles, the induction of paired box 7 (Pax7) was higher and stayed high until 4 days post-injury in Epsti1 KO muscles, implying that MuSCs in Epsti1 KO mice exhibit prolonged activation (Figure 2E). Despite the increase in Pax7 mRNA level, the mRNA levels of myogenin (MyoG) and embryonic myosin heavy chain (eMyHC) were reduced in Epsti1 KO mice. Additionally, the gene expressions of pro-inflammatory cytokines (TNF*α*, IL-6, and IL-1*β*) were significantly increased in Epsti1 KO mice (Figure 2F). These findings suggest Epsti1, an interferon response gene, plays a crucial role in limiting proinflammatory responses at the early stage of muscle regeneration.

### Epsti1-deficient myoblasts exhibited decreased myogenesis with increased STAT1 signaling

**Figure 3:**
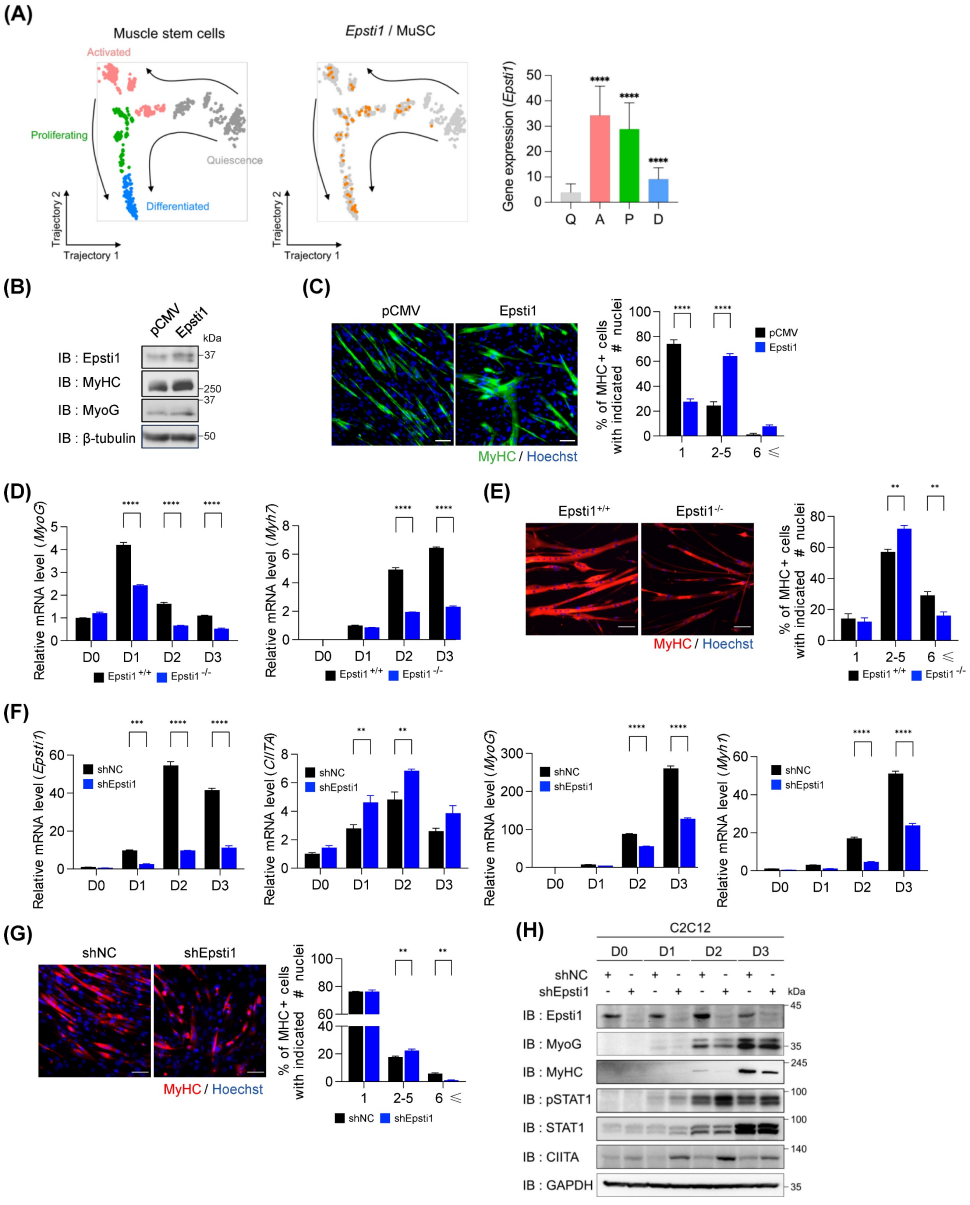
** Epsti1-deficient myoblasts exhibited decreased myogenesis with increased STAT1 signaling.** (A) Pseudotime analysis of myogenic cells (including Pax7+MuSCs, myogenic progenitors, and myoblasts) was performed by using monocle2, which revealed four different cell states. Expression of Epsti1 during muscle regeneration stages was visualized in orange (GSE150336) (left panel). Bar graph showing the expression of Epsti1 in each regeneration stage (Q, quiescence; A, activated; P, proliferating; D, differentiated) (right panel). (B) Immunoblot analysis of pCMV (control) and Epsti1 overexpressed C2C12 cells after 3 days of differentiation. (C) Immunofluorescence staining of MyHC in differentiated myotubes from pCMV and Epsti1 overexpressed C2C12 cells. Nuclei were stained with Hoechst. Quantification of MyHC-positive cells with the indicated number of nuclei is shown in the right panel. Scale bars, 30μm, *n* = 6 fields. (D) Relative mRNA levels of MyoG and Myh7 in Epsti1^+/+^ (WT) and Epsti1^-/-^ (Epsti1 KO) primary myoblasts at the indicated day of differentiation. N.D., not detected. *n* = 3. (E) Immunofluorescence staining of MyHC in differentiated myotubes from WT and Epsti1 KO primary myoblasts. Nuclei were stained with Hoechst. Quantification of MyHC-positive cells with the indicated number of nuclei is shown in the right panel. Scale bars, 30μm, *n* = 3 fields. (F) Relative mRNA levels of Epsti1, CIITA, MyoG, and Myh1 in control and Epsti1 knockdown C2C12 myoblasts at the indicated day of differentiation, *n* = 3. (G) Immunofluorescence staining of MyHC in differentiated myotubes from control and Epsti1 knockdown C2C12 myoblasts. Nuclei were stained with Hoechst. Quantification of MyHC-positive cells with the indicated number of nuclei is shown in the right panel. Scale bars, 30μm, *n* = 3 fields. (H) Immunoblot analysis of control and Epsti1 knockdown C2C12 cells at the indicated day of differentiation. In (A), data are presented as mean ± S.D. The statistical analysis was performed using one-way ANOVA. In (D), (E), (F), and (G), data are presented as mean ± S.E.M. The statistical analyses were performed using two-way ANOVA. **p<0.01, ***p<0.001, and ****p<0.0001.

To gain a deeper understanding of the transcriptional features of myogenic cells and their dynamics during the process of regeneration, we performed unsupervised clustering and pseudotime analysis on the myogenic cells using publicly available data (GSE150336). Based on their distinctive gene expression profiles, we labeled four subclusters: quiescent and activated MuSCs, proliferating myogenic progenitors, and differentiated myoblasts (Figure 3A and Figure S3A). In the pseudotime trajectory, the quiescent subcluster was plotted at the initial stage, with a branch consisting of a subset of activated MuSCs. Subsequently, the activated subcluster was fated to proliferate and differentiate. Notable, the gene expression of Epsti1 was enriched in the activated and proliferating subclusters. This finding suggests a potential involvement of Epsti1 in initiating myogenic differentiation. Therefore, we asked whether Epsti1 increases myogenic differentiation capacity in C2C12 myoblasts. Epsti1 overexpressing C2C12 cells displayed an increase in the expression of MyHC and MyoG proteins, and formed more larger myotubes than control C2C12, suggesting for a positive role of Epsti1 in myoblast differentiation (Figure 3B and C).

Next, we analyzed the myogenic potential of primary myoblasts. Compared to WT cells, the differentiating Epsti1 KO cells showed reduced transcription levels of myogenic genes (Figure 3D) and smaller myotubes (Figure 3E). Additionally, the knockdown of Epsti1 in C2C12 myoblasts (KD) using lentiviral short hairpin RNA (shRNA) also displayed a decreased differentiation potential compared to control cells (Figure 3F-H and Figure S3B and C).

Previous studies have shown that STAT1 signaling modulates the early stage of myogenesis by expressing MHC class II transactivator (CIITA) 11, 23, 24. CIITA interacts with MyoG and inhibits it to act as a transcription factor of muscle-specific genes, resulting in the suppression of myogenesis. Interestingly, Epsti1 KD myoblasts exhibited increased levels of phosphorylated STAT1 (pSTAT1) followed by an increase in gene activation and protein expression of CIITA (Figure 3H). These findings imply that the impaired differentiation of Epsti1-depleted myoblasts is attributable to dysregulation of the interferon response.

### VCP mediates the proteasomal degradation of IFN-γ-activated STAT1

**Figure 4:**
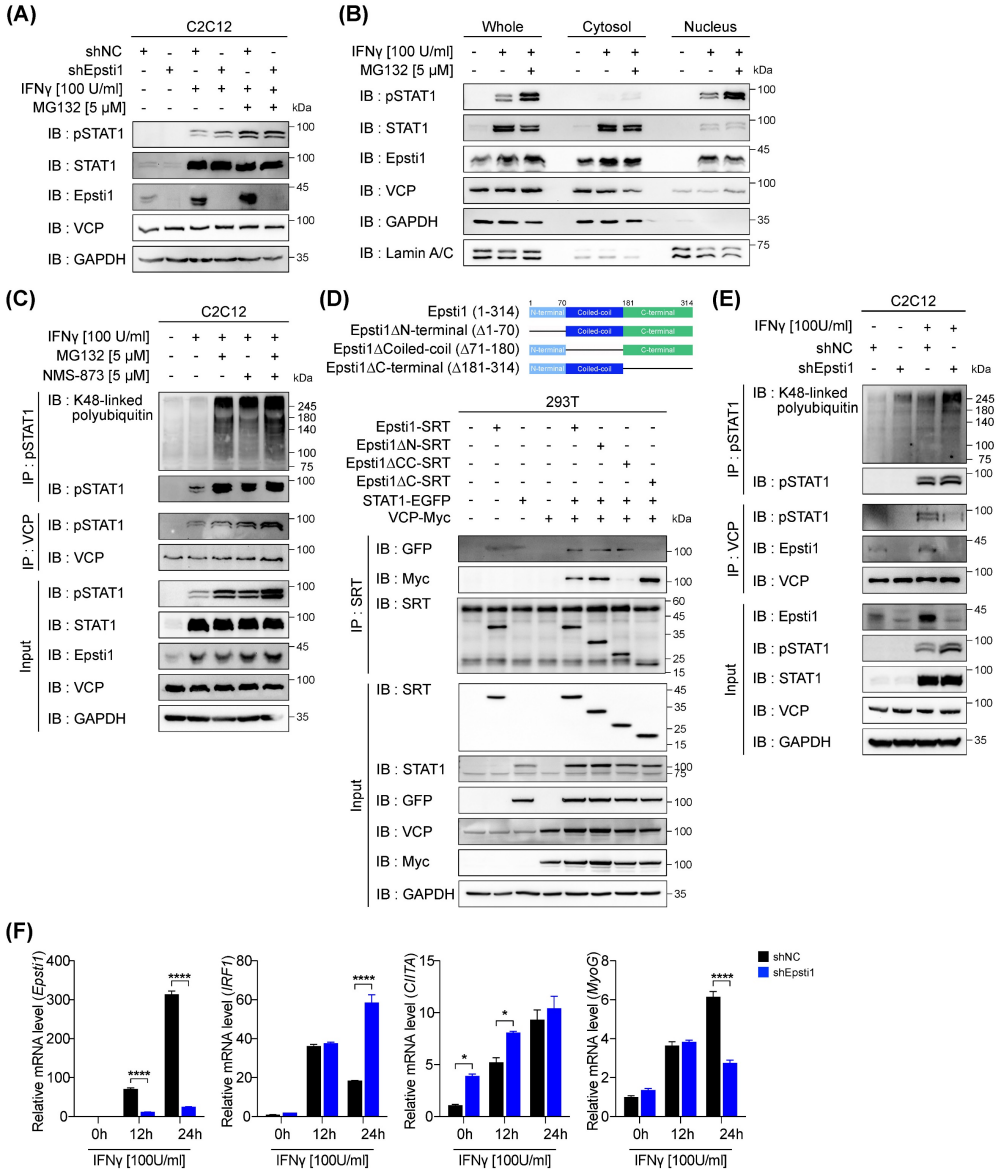
** Epsti1 regulates VCP-mediated degradation of IFN-γ-activated STAT1.** (A) Immunoblot analysis of in control (shNC) and Epsti1 knockdown (shEpsti1) C2C12 cells treated with IFN-γ (100 U/ml) for 24 hours and MG132 (5 µM) for the last 6 hours of the IFN-γ treatment. (B) Immunoblot analysis of cellular fractions isolated from C2C12 cells treated with IFN-γ (100 U/ml) for 24 hours and MG132 (5 µM) for the last 6 hours of the IFN-γ treatment. (C) Co-immunoprecipitation (Co-IP) analysis of C2C12 cells treated with IFN-γ (100 U/ml) for 24 hours, and treated with MG132 (5 µM) and/or NMS-873 (5 µM) for the last 6 hours of the IFN-γ treatment. (D) A schematic diagram representing the Epsti1 structural domains and its truncation mutants is shown in upper panel. Co-IP analysis of the interaction of VCP-Myc, STAT1-EGFP, Epsti1-SRT, and the Epsti1 truncation mutants in 293T cells. (E) Co-immunoprecipitation (Co-IP) analysis of in control (shNC) and Epsti1 knockdown (shEpsti1) C2C12 cells treated with IFN-γ (100 U/ml) for 24 hours. (F) Relative mRNA levels of Epsti1, IRF1, CIITA, and MyoG in control (shNC) and Epsti1 knockdown (shEpsti1) C2C12 cells treated with IFN-γ (100 U/ml) for the indicated time. In (F), data are presented as mean ± S.E.M. *n* = 3 independent biological experiments. The statistical analyses were performed using two-way ANOVA. *p<0.05 and ****p<0.0001.

Next, we focused on the underlying mechanism for the elevated levels of pSTAT1 in Epsti1 KD myoblasts. According to previous studies, it has been reported that pSTAT1 is degraded by the ubiquitin-proteasome system (UPS) 14. To investigate whether the elevated levels of pSTAT1 in Epsti1 KD cells resulted from decreased degradation, we inhibited the proteasomal degradation by MG132 treatment, following IFN-*γ* treatment. The treatment with MG132 led to an accumulation of pSTAT1 (Figure 4A). Interestingly, the accumulated levels of pSTAT1 were comparable between WT and Epsti1 KD cells. These results suggest that there is no significant difference in STAT1 phosphorylation between WT and Epsti1 KD cells. Moreover, these findings indicate a potential involvement of Epsti1 in pSTAT1 degradation. To determine the cellular localization for the degradation of pSTAT1, we performed cell fractionation analysis. The results demonstrated that pSTAT1 was exclusively found in the nucleus, and there was a notable accumulation of pSTAT1 within the nuclear compartment upon MG132 treatment (Figure 4B). Additionally, there was an augmented nuclear localization of Epsti1 following IFN-γ treatment. These results imply that the nucleus is the primary site of pSTAT1 degradation. Considering that pSTAT1 functions as a transcription factor by binding to the promoter of target genes, we postulated that a dissociation factor, such as VCP, is indispensable for the degradation process. VCP, also called AAA ATPase p97, acts as a segregase or unfoldase playing a key role in numerous ubiquitin-dependent pathways 25-28. It forms a homo-hexameric complex that interacts with a variety of cofactors and extracts ubiquitinated proteins from lipid membranes, chromatin, and protein complexes. It is to be noted that VCP was found in both cytosolic as well as nuclear fraction and nuclear VCP was increased upon MG132 treatment (Figure 4B), suggesting a potential involvement of VCP in the pSTAT1 degradation. We examined the level of IFN-γ-activated STAT1 following the cotreatment with MG132 and VCP inhibitor (NMS-873) (Figure 4C). The treatment of MG132 or NMS-873 resulted in an accumulation of ubiquitinated pSTAT1 proteins. Thus, the activity of VCP is also required for proteasomal degradation of ubiquitinated pSTAT1. These findings suggest that VCP mediates the proteasomal degradation of pSTAT1 in muscle cells.

### Epsti1 regulates the interaction between VCP and IFN-γ-activated STAT1

VCP is known to require a set of cofactor proteins to recognize target substrates 26, 28, 29. A previous study has suggested that Epsti1 can act as a cofactor of VCP by interacting with the 1-187 amino acids (aa) region of VCP 20. To further examine the binding domain of Epsti1 responsible for its interaction with VCP, we generated deletion constructs based on its domain structure (Figure 4D). The coiled-coil domain deletion (△71-180) of Epsti1 failed to immunoprecipitate with VCP. Furthermore, we confirmed that Epsti1 not only interacts with VCP but also with STAT1, and the C-terminal deletion (△181-314) of Epsti1 led to decreased binding with STAT1. These data suggest that the coiled-coil and C-terminal domains are required for the formation of Epsti1, VCP, and STAT1 complex.

Next, we analyzed the interaction between VCP and pSTAT1 in control and Epsti1 KD C2C12 cells in response to IFN-γ treatment. Knockdown of Epsti1 reduced the interaction between VCP and pSTAT1, which correlated with the increased accumulation of ubiquitinated pSTAT1 in Epsti1 KD cells compared to control cells (Figure 4E). Consistent with the elevated pSTAT1 levels in Epsti1 KD myoblasts, the expression of its target genes such as IFN-regulatory factor 1 (IRF1) and CIITA significantly increased in Epsti1 KD cells compared to the control cells in response to IFN-γ treatment (Figure 4F). In contrast to the increased levels of CIITA, the expression of MyoG was significantly reduced in Epsti1 KD cells. Collectively, these results demonstrate that Epsti1 functions as a crucial regulator in the VCP-mediated degradation of IFN-γ-activated STAT1.

### Epsti1 deficiency aggravates muscle wasting in a cancer cachexia model

**Figure 5:**
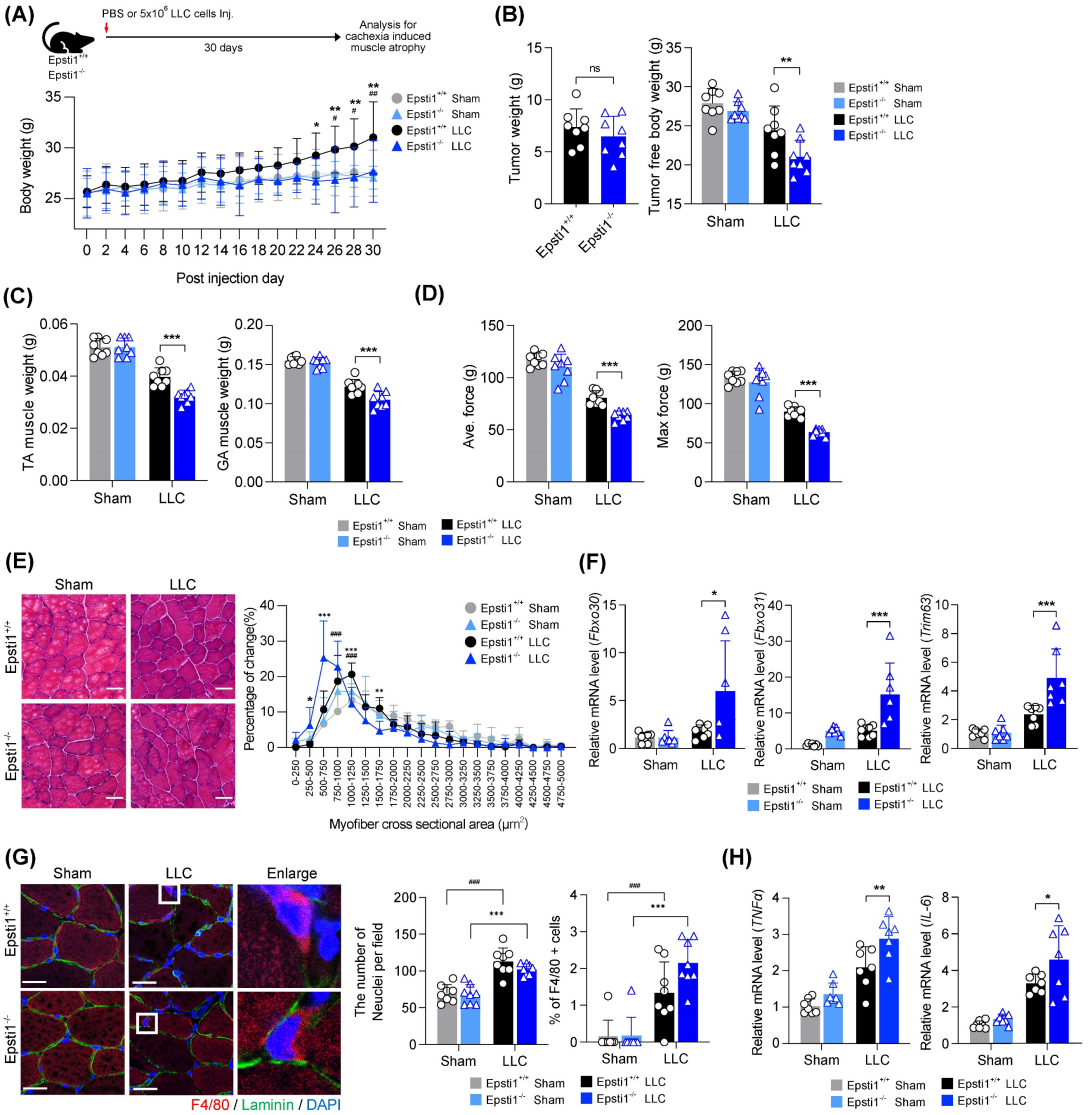
** Mice lacking Epsti1 exhibited more severe muscle atrophy with excessive inflammation in cancer cachexia.** (A) Experiment schematic of Lewis lung carcinoma (LLC) induced cachexia model and average body weight of Epsti1^+/+^ (WT) and Epsti1^-/-^ (Epsti1 KO) mice after sham or LLC tumor graft. Mice were sacrificed at 30 days after PBS or LLC cells injection. (B) Tumor mass and tumor-free body weight of WT and Epsti1 KO sham or LLC-bearing mice. (C) Weight of dissected tibialis anterior (TA) and gastrocnemius (GA) muscles. (D) Average and maximum force of muscle grip strength. (E) Representative images of H&E-stained TA sections. Quantification of myofiber cross-sectional areas (CSA) in TA muscles is shown in the right panel. Scale bars, 30μm. (F-G) Relative mRNA levels of Fbxo30, Fbxo31, Trim63, TNF*α*, IL-6, and IL-1*β* from TA muscles. (H) Representative images of immunostaining for F4/80 and laminin with TA sections from WT and Epsti1 KO sham or LLC-bearing mice. Nuclei were stained with DAPI. Quantification of the number of nuclei or % of F4/80 positive cells is shown in the right panel. Scale bars, 25μm. All data are presented as mean ± S.D. *n* = 7-8 mice per group. The statistical analyses were performed using two-way ANOVA except for the analysis of tumor weight in (B) which was performed using two-tailed Student's t-test. ^#^p<0.05, ^##^p<0.01, and ^###^p<0.001 (WT sham vs. WT LLC); *p<0.05, **p<0.01, and ***p<0.001 (WT LLC vs. Epsti1 KO LLC); ns, not significant.

Based on above mentioned role of Epsti1 in inflammatory response, Epsti1-deficient mice are predicted to have a poor prognosis in cachexia, which is accompanied by excessive inflammation and muscle atrophy. To validate this hypothesis, we induced cachexia by injection of Lewis lung carcinoma (LLC) cells into WT and Epsti1 KO mice (Figure 5A). The body weights of cachectic mice were increased during the progression of tumor formation. However, the body weights of Epsti1 KO cachectic mice were lower than that of WT cachectic mice after 24 days, suggesting an early onset of cachexia-induced muscle wasting. There were no significant differences in the weights of tumor, white adipose tissue, spleen, heart, and liver between WT and Epsti1 KO cachectic mice (Figure 5B and Figure S4A). However, Epsti1 KO cachectic mice exhibited declines in tumor-free body weights and the weights of TA and GA muscles compared to those of WT cachectic mice (Figure 5B, C, and Figure S4B). Along with the reduced muscle weights, the average and maximal grip force and muscle cross-sectional areas were much smaller in Epsti1 KO cachectic mice than in WT cachectic mice (Figure 5D and E). Moreover, atrophy-related genes (Fbxo30, Fbxo31, and Trim63) and inflammatory-related genes (TNFα and IL-6) were significantly upregulated in Epsti1 KO cachectic muscles than in WT cachectic muscles (Figure 5F, G, and Figure S4C). To address the muscle inflammation, we analyzed macrophage infiltration in each group. The number of nuclei was increased in cachectic muscles, compared to sham muscles, suggesting for an alteration of muscle resident cells, such as macrophage or fibroblasts. Consistently, macrophages were undetectable in sham muscles of both WT and Epsti1 KO mice while cachectic muscles of WT and Epsti1 KO mice exhibited increased macrophage recruitment (Figure 5H). However, these was no difference between cachectic muscles. These results suggested that Epsti1 deficiency increases the sensitivity to inflammatory signals in muscle. In summary, our data demonstrate that Epsti1 plays a critical role in muscle regeneration and the prevention of cachexia-induced muscle wasting through limiting inflammatory responses by modulation of the VCP/STAT1 axis.

## Discussion

In the current study, we demonstrate the crucial role of Epsti1 in muscle regeneration and prevention of muscle wasting from cancer cachexia. Epsti1 depletion causes enhanced STAT1 activity attributing to excessive inflammation leading to impaired muscle regeneration. Epsti1 deficiency resulted in enhanced expression of Pax7 along with elevated levels of pro-inflammatory cytokines. In addition, Epsti1 deficiency led to a great decline in the expression of the myogenic differentiation markers MyoG and eMyHC. These data demonstrate that Epsti1 deficiency inhibits the transition of activated muscle stem cells from a proliferating state to a differentiation state, which is essential to complete muscle repair. Epsti1 is specifically induced in muscle stem cells upon injury, suggesting for its role to modulate inflammatory responses in muscle stem cells. Consistently, Epsti1 depletion resulted in enhanced levels of STAT1 activation attributable to inhibition of myoblast differentiation. These results are in line with the previous report demonstrating the effect of STAT1 in the prevention of myoblast differentiation mediated by JAK1 30. Sun et al. have proposed the significance of the JAK1/STAT1 pathway in inhibiting premature differentiation of myoblasts, thereby ensuring effective muscle regeneration. The balanced regulation between pro-inflammatory and anti-inflammatory signaling is nodal for the proper progression of muscle regeneration 31, 32. Thus, the counteractive effect of Epsti1 in preserving STAT1 activity could have a crucial role in driving MuSCs from proliferation to the differentiation process. Consistently, the induction of Pax7 was elevated and sustained in Epsti1 KO muscles until day 4 post-injury, while it started to decline at day 4 post-injury in WT muscles. In contrast, the expression levels of early differentiation markers, MyoG and eMyHC exhibited a significant decrease.

Besides Epsti1-dependent pSTAT1 degradation, the suppressor of cytokine signaling (SOCS) family of proteins plays a negative regulator for cytokine-induced JAK/STAT signaling 33. Among the eight SOCS family of proteins, SOCS3 is the best studied in muscle 34, 35. SOCS3 exhibits a high expression in quiescent muscle stem cells, suggesting for its role in stem cell quiescence 36. In addition, SOCS3 has been shown to induce myogenic differentiation capacity in C2C12 myoblasts 37. Thus, it is considered to regulate muscle regeneration process. In our current study, we show that SOCS3 is highly expressed at 0.5 DPI in muscle (Figure 1F). However, previous studies reported no alteration in muscle regeneration capacity in myofiber-specific and satellite cell specific SOCS3 knockout mice 38, 39. Thus, the involvement of SOCS3 to restrain the JAK/STAT signaling in the early muscle regeneration seems to be not the major mechanism and needs to be further investigated.

Macrophages exert trophic roles on MuSCs and other cell types within the muscle following injury 31, 40, 41. A previous study has shown that Epsti1-deficient bone marrow-derived macrophages exhibit enhanced M2-type macrophage phenotype 21. However, the specific role of Epsti1 in muscle resident macrophages remains unknown. Although muscle resident macrophages are heterogeneous, and the interactions between MuSCs and macrophages are complex, it has been suggested that the shift from M1 to M2 macrophage phenotypes could influence the transition of proliferating MuSCs into differentiating muscle progenitors 42-44. Our current findings indicate that Epsti1 KO muscles exhibit enhanced and prolonged expression of proinflammatory cytokines, which are attributable to hindering the progression of muscle differentiation. Nevertheless, further investigations are required to fully understand the precise role of Epsti1 in muscle resident macrophages.

**Figure 6:**
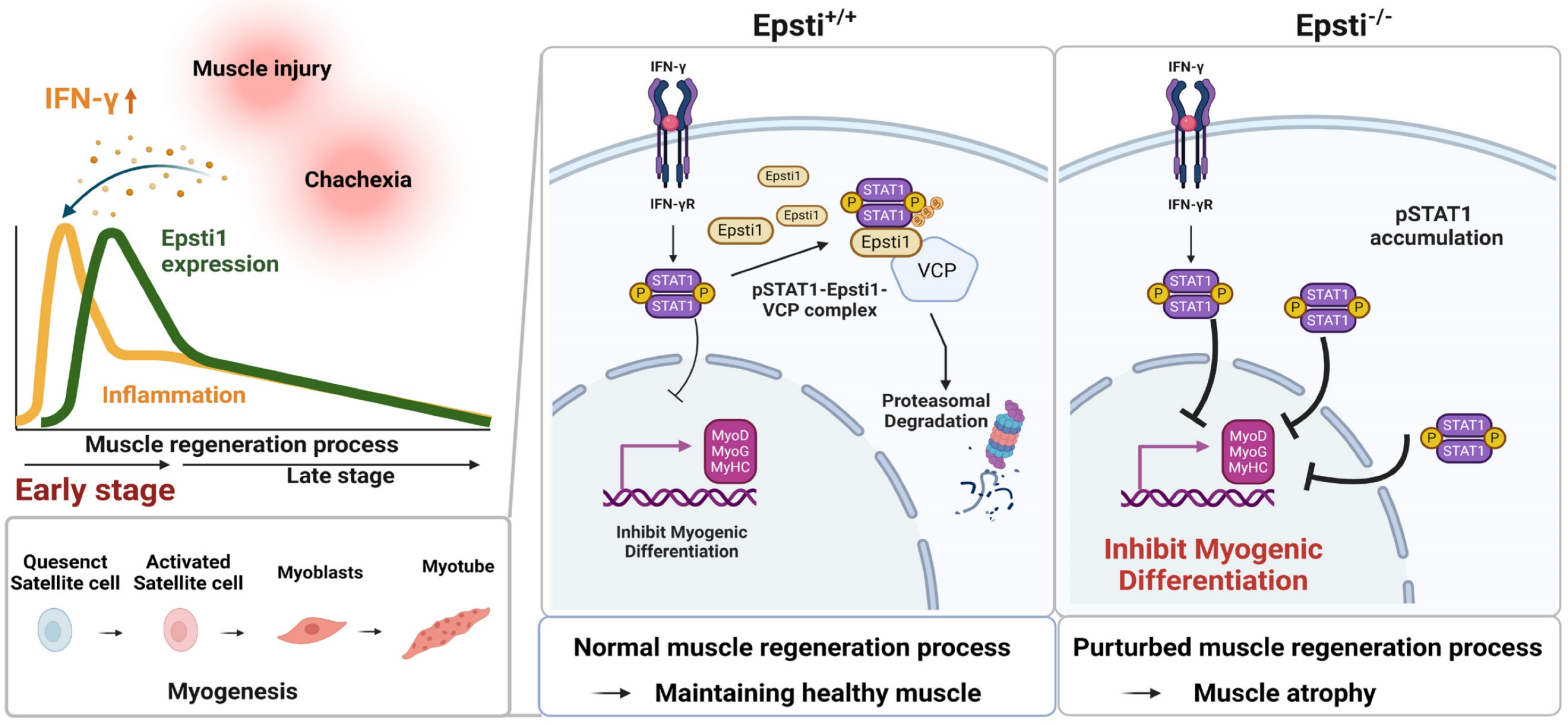
** Schematic diagram illustrating the mechanism of degradation of IFN-γ-activated STAT1 through VCP-Epsti1 complex.** In the early stages of muscle regeneration, the activation of STAT1 by IFN-γ in myogenic cells leads to the upregulation of inflammatory cytokines and the concurrent suppression of the expression of myogenic genes. In this context, Epsti1 plays a regulatory role in facilitating VCP-mediated proteasomal degradation of IFN-γ-activated STAT1 during muscle regeneration. The consequent resolution of IFN-γ-STAT1 signaling is critical for the transition from the inflammatory stage to the myogenesis stage for maintaining muscle regeneration capacity.

Diverse mechanisms including phosphorylation and acetylation can modulate the activity of STAT1 45, 46. Additionally, the ubiquitination of pSTAT1 appears to be critical to terminate the interferon responses 14. When proteasomal degradation is blocked in myoblast, the levels of pSTAT1 strongly accumulate in the nucleus. One key regulator responsible for the degradation of pSTAT1 is a segregase VCP, which facilitates the extraction of ubiquitinated proteins for proteasomal degradation 28. VCP requires various cofactors to recognize the target substrate 26-28, and Epsti1 has been suggested to act as a cofactor of VCP 20. Our current data demonstrate that Epsti1 physically interacts with VCP through its N- and C-terminal domains. It indicates that Epsti1 likely acts as a cofactor of VCP to modulate the interaction between VCP and pSTAT1, which is critical for pSTAT1 degradation. Consequently, depletion of Epsti1 leads to an increase in the accumulation of K48-linked ubiquitination of pSTAT1, concurrent with elevated STAT1 signals. Inflammation is a common risk factor for diverse muscle wasting conditions, including cancer cachexia-mediated muscle wasting 47. Consistently, Epsti1-deficient mice exhibit more severe muscle wasting in the cancer cachexia model, strongly underscoring Epsti1 as an important modulator of inflammatory responses in muscle maintenance. Moreover, human studies have revealed that the expression of Epsti1 is upregulated in aged individuals who are more likely to have chronic inflammation compared to younger counterparts, suggesting that Epsti1 may also regulate inflammatory responses in humans (Figure S4D). Taken together, our current study demonstrates a novel function of Epsti1 in modulation of inflammatory responses in myoblasts required for the efficient muscle regeneration (Figure 6). Our findings provide valuable insights into understanding the mechanism underlying the resolution of IFN-γ-JAK-STAT1 signaling during the early stages of muscle regeneration.

## Supplementary Material

Supplementary images, figures and tables.

## Abbreviations

IFN-*γ*: Interferon-gamma
JAK: Janus kinase
STAT1: Signal transducer and activator of transcription 1
Epsti1: Epithelial-stromal interaction 1
VCP: Valosin-containing protein
MPCs: Myogenic precursor cells
UPS: ubiquitin-proteasome system
MuSCs: Muscle stem cells
PID: Days post-injury
CTX: Cardiotoxin
Tnf: Tumor necrosis factor
Il6: Interleukin 6
Pax7: Paired box 7
MyoG: Myogenin
eMyHC: Embryonic myosin heavy chain
CIITA: MHC class II transactivator
IRF1: IFN-regulatory factor 1
LLC: Lewis lung carcinoma
Fbxo30: F-box protein 30
Fbxo31: F-box protein 31
Trim63: Tripartite motif-containing 63
SOCS: Suppressor of cytokine signaling
