# Supplementary material for: Epsti1 Regulates the Inflammatory Stage of Early Muscle Regeneration through STAT1-VCP Interaction
Source: Int J Biol Sci. 2024 Jun 24;20(9):3530–43. doi: 10.7150/ijbs.94675 (PMC11234217; doi:10.7150/ijbs.94675)
Supplement: Supplementary file 1 — Supplementary images, figures and tables. [file ijbsv20p3530s1.pdf]

Supplementary material

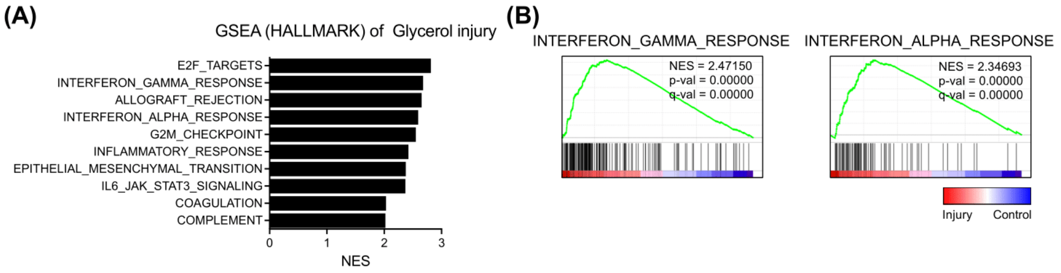

(C)

GENE SET	PROBE	DESCRIPTION	RANK IN GENE LIST	RANK METRIC SCORE	RUNNING ES	CORE ENRICHMENT
Interferon gamma response	EPSTI1	epithelial stromal interaction 1 [Source:MGI Symbol;Acc:MGI:1915168]	1242	2.96450	0.40697	Yes
Interferon alpha response					0.39207	Yes

Figure S1. Gene set enrichment analysis of injured muscles by glycerol

(A) Top 10 Hallmark biological processes enriched in muscles at 3 days after glycerol injury.

(B) Enrichment plots of interferon-gamma response and interferon alpha response gene set in glycerol-injured muscles.

(C) Information that Epsti1 belongs to the leading subset of the interferon-gamma and interferon alpha response gene sets in glycerol-injured muscles.

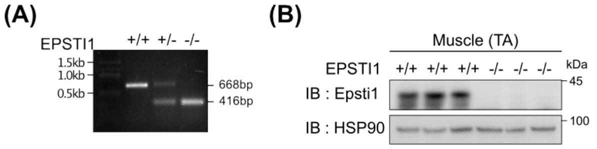

Figure S2. Analysis of gene and protein expression of Epsti1 in WT and Epsti1 KO mice

(A) Genotype identification of Epsti1$^{+/+}$ (WT), Epsti1$^{+/-}$, and Epsti1$^{-/-}$ (Epsti1 KO) mice.

(B) Immunoblot analysis of Epsti1 and HSP90 in TA muscles of WT and Epsti1 KO mice. n = 3 mice per group.

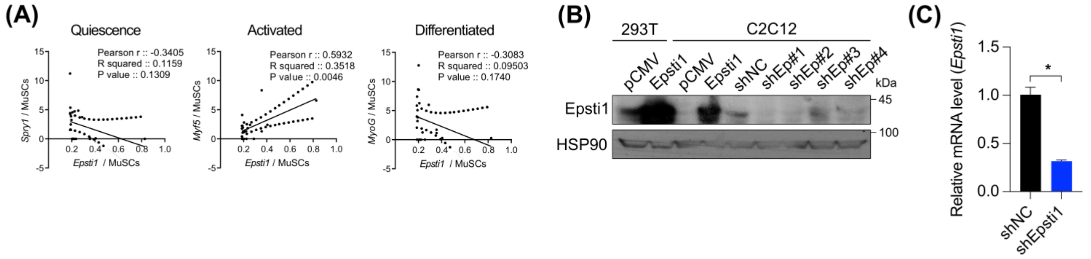

Figure S3. Correlation analysis of Epsti1 with myogenic markers and validation of Epsti1 knockdown in C2C12 cells

(A) Correlation scatterplot of Epsti1 and myogenic development markers (Quiescence, Spry1; Activated, Myf5; Myoblast, MyoG) in MuSCs from single-cell RNA profiling of CTX injured mice (GSE150366).

(B) Immunoblot analysis of Epsti1 and HSP90 derived from 293T and C2C12 cells. For overexpression of Epsti1, 293T and C2C12 cells were transfected with pCMV and Epsti1. For the knockdown of Epsti1, C2C12 cells were transduced with lentiviral shNC and shRNAs targeting Epsti1 (shEpsti1 #1~#4).

(C) Relative mRNA levels of Epsti1 in control (shNC) and Epsti1 knockdown (shEpsti1) C2C12 with selected shRNA (shEpsti1 #1). The statistical analysis in (C) was performed using one-tailed Student's t-test. $^{*}p<0.05$

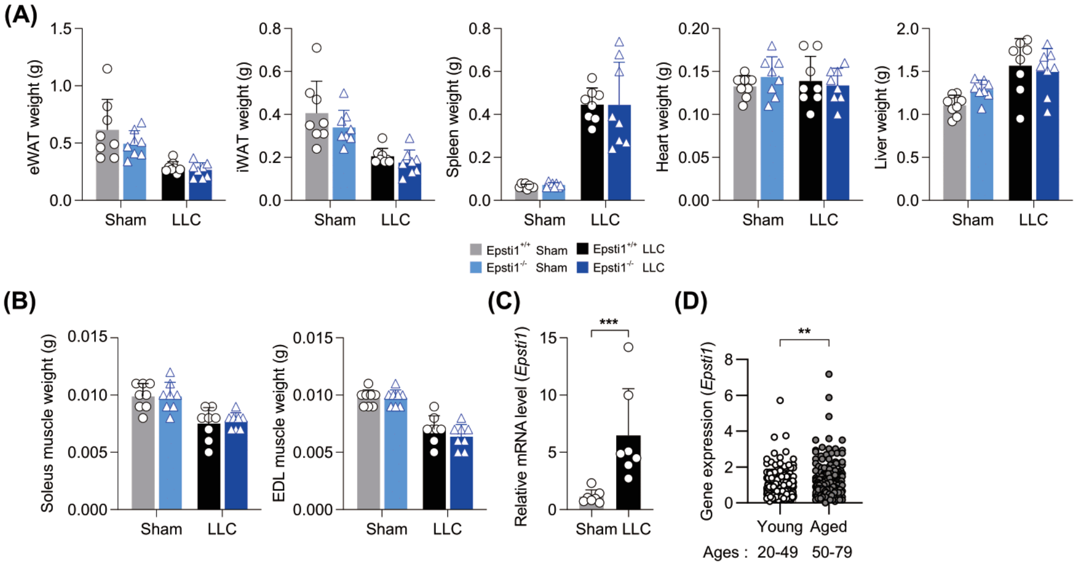

Figure S4. Epsti1 KO mice exhibited more severe muscle atrophy in cancer cachexia

(A) Weight of dissected organs (eWAT, iWAT, spleen, heart, and liver).

(B) Weight of dissected soleus and extensor digitorum longus (EDL) muscles.

(C) Relative mRNA levels of Epsti1 from TA muscles.

(D) Relative mRNA levels of Epsti1 from skeletal muscles of young (20-49 years, $n = 256$) and aged (50-79 years, $n = 291$) humans.

In (A), (B), and (C), data are presented as mean ± S.D. $n = 8$ mice per group. The statistical analyses were performed using two-way ANOVA except for the analyses in (C) and (D) which were performed using two-tailed Student's t-test. **p<0.01, ***p<0.001.

Table S1. Primer sequence of genes

Gene	Sequences	
	Forward	Reverse
Epsti1 (for genotyping)	ACC AGC TCA GTT CTT CAG CC	GAA TCA GTG GAC TGG GGG TG
Epsti1	CTT CCA GTC CTG CTT AGA GAT C	TGG TGC TGT CCT TTA GTT TCC
Pax7	CCG TGT TTC TCA TGG TTG TG	GAG CAC TCG GCT AAT CGA AC
MyoD	AGT GAA TGA GGC CTT CGA GA	GCA TCT GAG TCG CCA CTG TA
MyoG	ATC TCC GCT ACA GAG GCG GG	TAG GGT CAG CCG CGA GCA AA
eMyHC	CCT TCT GGA GCA GGA CAG AA	CAA AGC AAA GTT TAT TGC ATG TG
Myh7	CCA TCT CTG ACA ACG CCT ATC	GGA TGA CCC TCT TAG TGT TGA C
TNFα	ACT GGC AGA AGA GGC ACT CC	GCC ACA AGC AGG AAT GAG AA
IL-6	GAT AAG CTG GAG TCA CAG AAG G	GAA ACC ATC TGG CTA GGT AAC A
IL-1β	CCA GGA TGA GGA CAT GAG CA	CGG AGC CTG TAG TGC AGT TG
IRF1	ATA ACT CCA GCA CTG TCA CCG TG	ATC CTC GTC TGT TGC GGC TT
CIITA	TTC ACC ATT GAG CCA TTT AAA GC	CTG GGT CTG CAC GAG ACG AT
Fbxo30	TCG TGG AAT GGT AAT CTT GC	CCT CCC GTT TCT CTA TCA CG
Fbxo31	CAT GCG GTT CAA GCC ACT G	GTC TGG TTA CAC TTG GTG GAG
Fbxo32	GAC CGG CTA CTG TGG AAG AG	CCA GGA GAG AAT GTG GCA GT
Trim63	GAG CAA GGC TTT GAG AAC ATG GAC T	GCG TCC AGA GCG TGT CTC ACT

Figure S5. Original images of western blot

Figure 1G

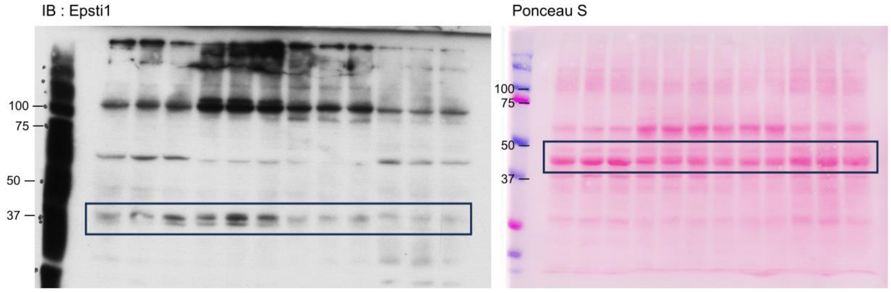

Figure 3B

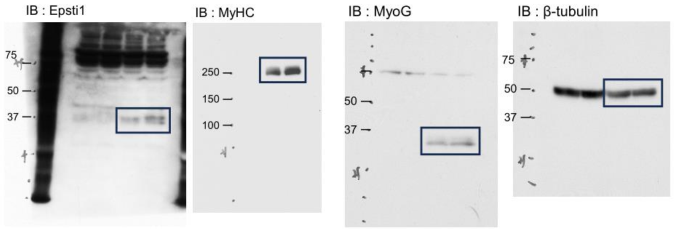

Figure 3H

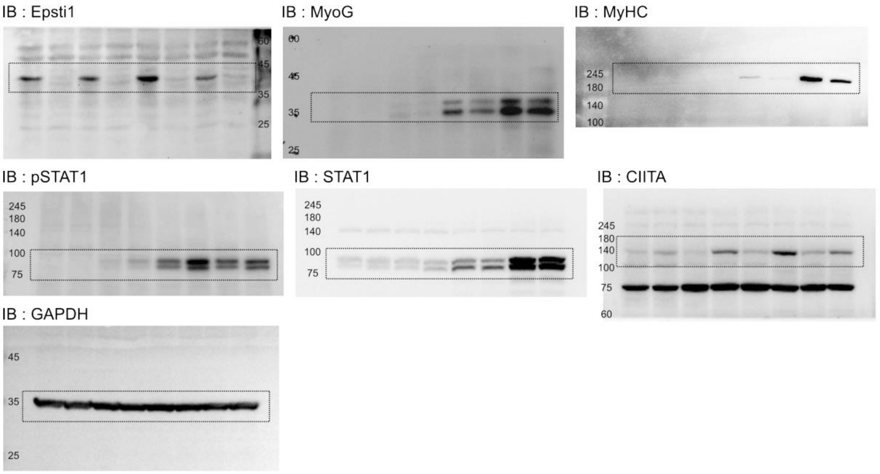

Figure 4A

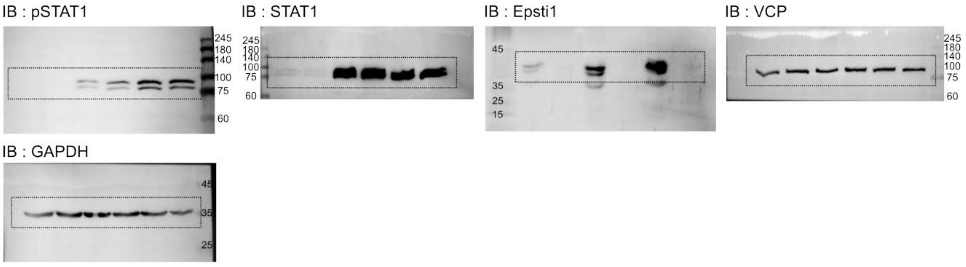

Figure 4B

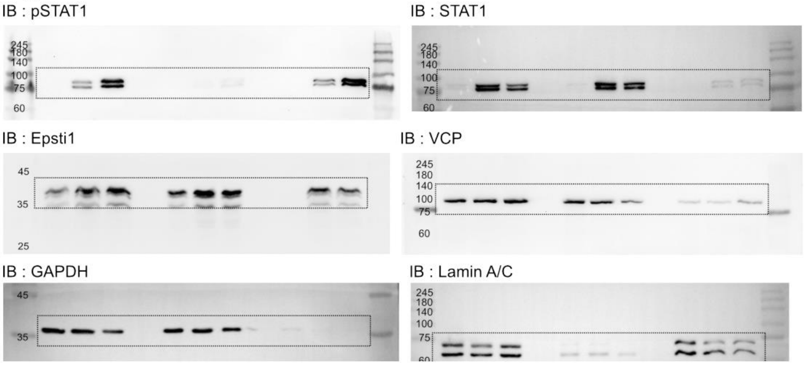

Figure 4C

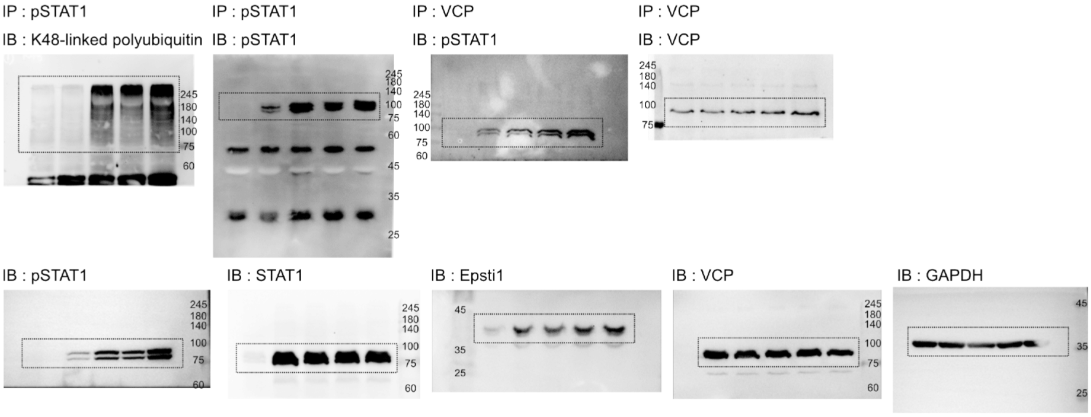

Figure 4D

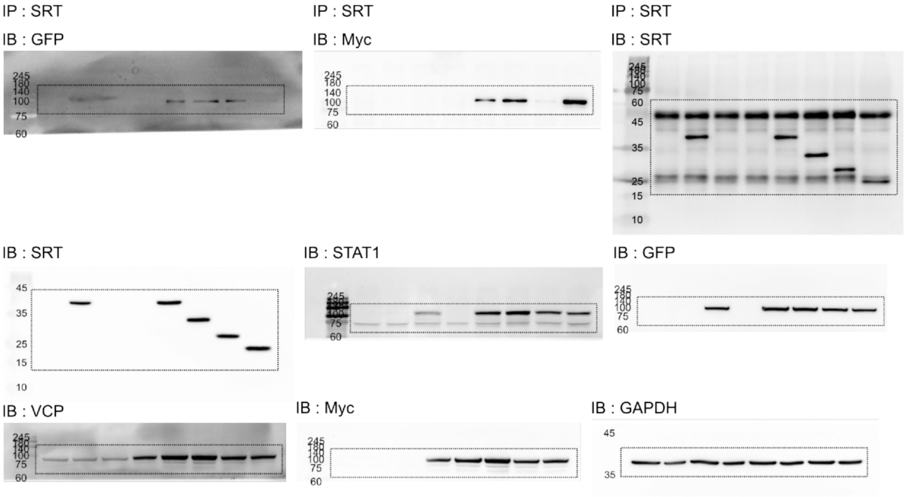

Figure 4E

IP : pSTAT1 | IP : pSTAT1 | IP : VCP | IP : VCP | IP : VCP

IB : K48-linked polyubiquitin | IB : pSTAT1 | IB : pSTAT1 | IB : Epsti1 | IB : VCP

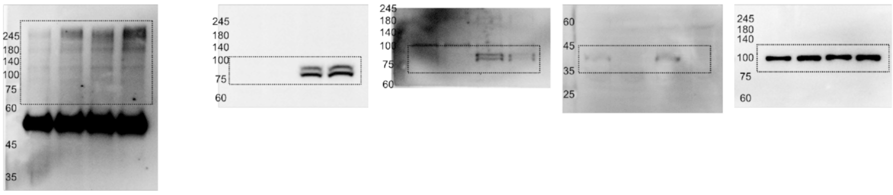

IB : Epsti1 | IB : pSTAT1 | IB : STAT1 | IB : VCP | IB : GAPDH

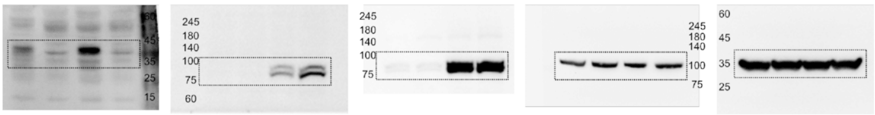

Supplementary Figure 2B

IB : Epsti1 | IB : HSP90

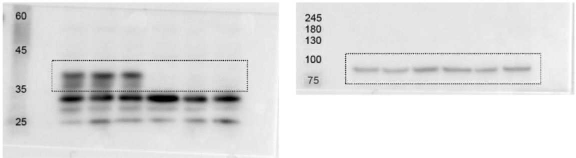

Supplementary Figure 3C

IB : Epsti1 | IB : HSP90

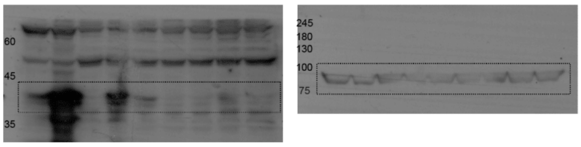

Table S1. Primer sequence of genes

Gene	Sequences	
	**Forward**	**Reverse**
Epsti1 (for genotyping)	ACC AGC TCA GTT CTT CAG CC	GAA TCA GTG GAC TGG GGG TG
Epsti1	CTT CCA GTC CTG CTT AGA GAT C	TGG TGC TGT CCT TTA GTT TCC
Pax7	CCG TGT TTC TCA TGG TTG TG	GAG CAC TCG GCT AAT CGA AC
MyoD	AGT GAA TGA GGC CTT CGA GA	GCA TCT GAG TCG CCA CTG TA
MyoG	ATC TCC GCT ACA GAG GCG GG	TAG GGT CAG CCG CGA GCA AA
eMyHC	CCT TCT GGA GCA GGA CAG AA	CAA AGC AAA GTT TAT TGC ATG TG
Myh7	CCA TCT CTG ACA ACG CCT ATC	GGA TGA CCC TCT TAG TGT TGA C
TNFα	ACT GGC AGA AGA GGC ACT CC	GCC ACA AGC AGG AAT GAG AA
IL-6	GAT AAG CTG GAG TCA CAG AAG G	GAA ACC ATC TGG CTA GGT AAC A
IL-1β	CCA GGA TGA GGA CAT GAG CA	CGG AGC CTG TAG TGC AGT TG
IRF1	ATA ACT CCA GCA CTG TCA CCG TG	ATC CTC GTC TGT TGC GGC TT
CIITA	TTC ACC ATT GAG CCA TTT AAA GC	CTG GGT CTG CAC GAG ACG AT
Fbxo30	TCG TGG AAT GGT AAT CTT GC	CCT CCC GTT TCT CTA TCA CG
Fbxo31	CAT GCG GTT CAA GCC ACT G	GTC TGG TTA CAC TTG GTG GAG
Fbxo32	GAC CGG CTA CTG TGG AAG AG	CCA GGA GAG AAT GTG GCA GT
Trim63	GAG CAA GGC TTT GAG AAC ATG GAC T	GCG TCC AGA GCG TGT CTC ACT

Table S2. Antibodies

Antibodies	Source	Catalog	Concentrations
Epsti1	Proteintech	11624-1-AP	IB: 1:500
SRT		-	IB: 1:5,000
Phospho-Stat1 (Tyr701)	Cell signaling	9167	IB: 1:1000
STAT1	Cell signaling	9172	IB: 1:1000<br>IP : 1:200
VCP	Santacruz	sc57492	IB: 1:1000<br>IP : 1:200
Myc	Bethyl	A190-104A	IB: 1:1000<br>IP : 1:200
Ubiquitin	Santacruz	sc8017	IB: 1:1000
K48-linkage specific polyubiquitin	Cell signaling	8081	IB: 1:1000
MyHC	DSHB	MF20 (hybridoma)	IB: (supernatant)
MyoG	DSHB	IF05 (hybridoma)	IB: (supernatant)
GAPDH	Santacruz	sc32233	IB: 1:10,000
HSP90	Santacruz	sc13119	IB: 1:10,000
HRP-linked anti-mouse IgG	Jackson Immunoresearch	715-035-150	IB : 1:5,000
HRP-linked anti-rabbit IgG	Jackson Immunoresearch	715-035-152	IB : 1:5,000
HRP-linked anti-goat IgG	Jackson Immunoresearch	715-035-003	IB : 1:5,000
eMyHC	DSHB	F1.652	IF: 1:200
F4/80	eBioscience	12-4801-82	IF: 1:200
Laminin	Abcam	ab11575	IF: 1:200
Alexa Fluor 488-linked anti-mouse IgG	Thermo Fisher	A28175	IF: 1:200
Alexa Fluor 555-linked anti-mouse IgG	Thermo Fisher	A28180	IF: 1:200
Alexa Fluor 555-linked anti-rabbit IgG	Thermo Fisher	A27039	IF: 1:200
